# Intranasal stem cell treatment ameliorates neuroinflammation and myelin aberrations in fetal growth-restricted rat pups

**DOI:** 10.1093/stcltm/szag081

**Published:** 2026-09-12

**Authors:** Judit Alhama-Riba, Anastasija Aleksić, Nicola Pelizzi, Freek E Hoebeek, Caroline G M de Theije, Cora H A Nijboer

**Affiliations:** Department for Developmental Origins of Disease (DDOD), Brain Center, University Medical Center Utrecht, Wilhelmina Children’s Hospital, Utrecht University, Utrecht, The Netherlands; Department for Developmental Origins of Disease (DDOD), Brain Center, University Medical Center Utrecht, Wilhelmina Children’s Hospital, Utrecht University, Utrecht, The Netherlands; Global R&D, Chiesi Farmaceutici S.p.A., Parma, Italy; Department for Developmental Origins of Disease (DDOD), Brain Center, University Medical Center Utrecht, Wilhelmina Children’s Hospital, Utrecht University, Utrecht, The Netherlands; Department for Developmental Origins of Disease (DDOD), Brain Center, University Medical Center Utrecht, Wilhelmina Children’s Hospital, Utrecht University, Utrecht, The Netherlands; Department for Developmental Origins of Disease (DDOD), Brain Center, University Medical Center Utrecht, Wilhelmina Children’s Hospital, Utrecht University, Utrecht, The Netherlands

**Keywords:** mesenchymal stem cells, intranasal therapy, fetal growth restriction, neurological outcome, reduced uterine perfusion pressure rat model

## Abstract

Fetal growth restriction (FGR), a condition in which a fetus fails to reach its full growth potential, affects approximately 30 million infants worldwide each year and increases the risk of long-term neurodevelopmental deficits. Currently, no effective therapies exist to mitigate FGR-induced neurological impairments. Mesenchymal stem cells (MSCs) have emerged as a promising neuroregenerative therapeutic approach. Here, we used the reduced uterine perfusion pressure (RUPP) rat model of chronic placental hypoperfusion-induced FGR. We explored, for the first time, intranasal MSC administration in the early postnatal period in growth-restricted offspring as a minimally invasive and clinically feasible cell delivery approach. RUPP pups received human Wharton’s jelly-derived MSCs or vehicle intranasally at postnatal day 5 (P5) or P10, and neuropathological outcomes were assessed at P20. Pups from sham-operated dams served as controls. Intranasal MSC treatment, particularly at the early postnatal treatment timepoint (P5), ameliorated key neuropathological outcomes, as evidenced by improved brain weight and cortical myelination, enhanced oligodendrocyte maturation, and dampened microglial activation compared to vehicle-treated RUPP pups. Functional impairments, measured between P13 and P56 by eye opening, grip strength, and foot faults on the tapered beam walk, showed no significant improvement. Our findings provide proof-of-concept that early-life stem cell therapy provides histological improvements in RUPP-induced brain abnormalities. Although further optimization is needed to establish whether these effects translate into functional benefits, intranasal MSCs represent a promising, minimally invasive neuroregenerative strategy with potential clinical relevance for newborns affected by chronic placental insufficiency-induced FGR.

## Graphical abstract

**Graphical Abstract:**
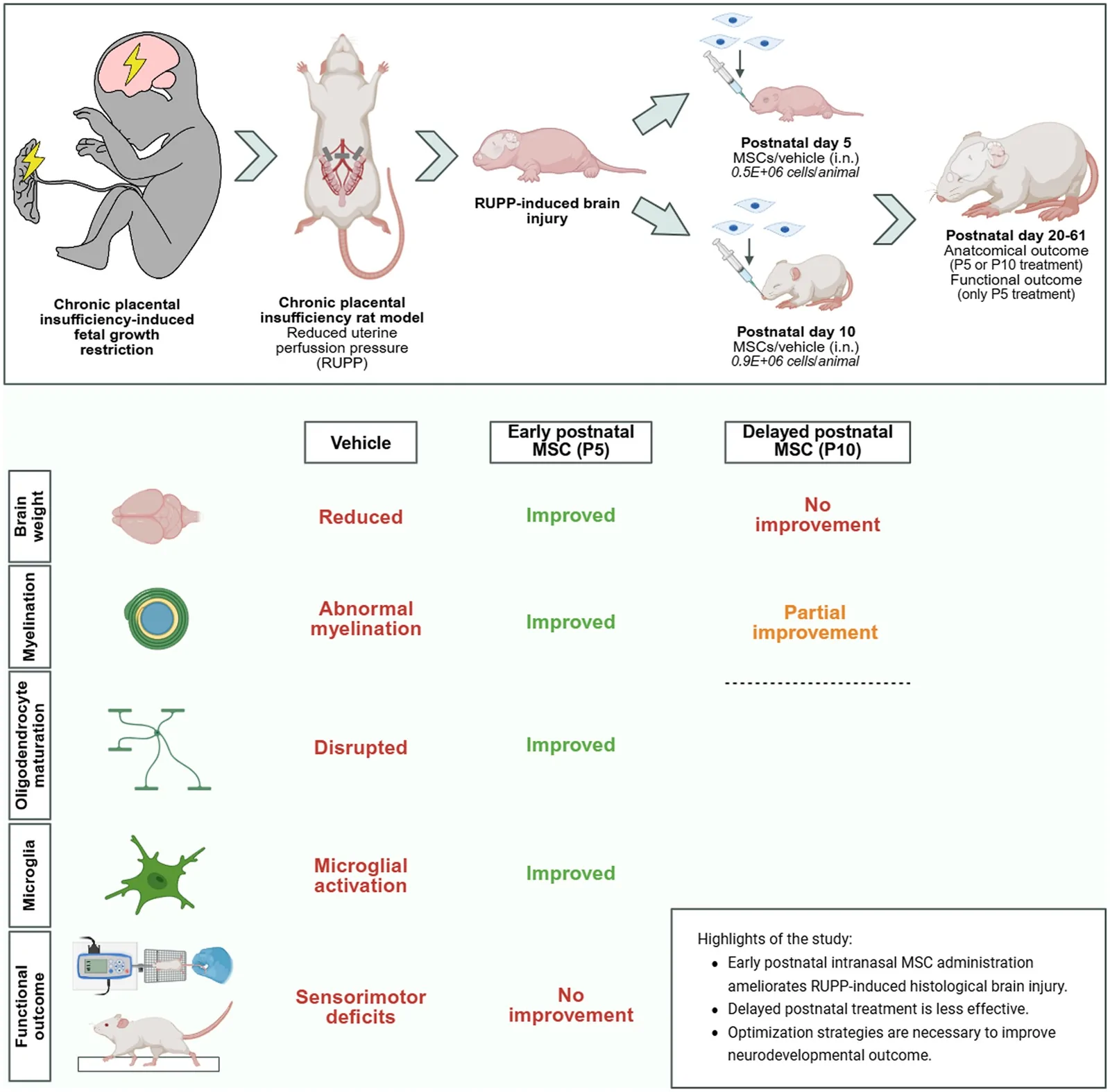

Significance statement Fetal growth restriction is a global health challenge with an impact on brain development. This study shows that intranasal stem cell administration can ameliorate several histological brain abnormalities in an established animal model of chronic placental insufficiency-induced fetal growth restriction. Postnatal stem cell treatment improved brain growth and myelination aberrations, supported oligodendrocyte maturation, and reduced neuroinflammation, but did not improve functional outcome. These findings provide proof-of-concept for a minimally invasive cell-based therapy with potential clinical relevance for affected newborns, while further optimization is required to establish functional benefit.

## Introduction

Fetal growth restriction (FGR), the failure of a fetus to achieve its growth potential, affects 3%-10% of pregnancies worldwide (∼30 million annually).^1–3^ Placental insufficiency, commonly caused by preeclampsia, is the leading cause of FGR, limiting oxygen and nutrient supply to the fetus and impairing fetal growth.^1^^,^^4^^,^^5^ Although fetal blood flow is redirected to the brain (brain-sparing), this adaptation provides only partial neuroprotection.^3^^,^^6^ Consequently, growth-restricted infants are at increased risk of long-term motor, cognitive, and behavioral impairments.^2^^,^^3^ Current therapies are limited; early induced delivery remains the only effective intervention for preeclampsia, resulting in preterm birth and associated neonatal risks.^1^^,^^7^^,^^8^

FGR markedly affects brain development, impacting both gray matter (GM) and white matter (WM).^6^ GM volume and cortical thickness are reduced antenatally and remain decreased into adulthood.^3^^,^^9^^,^^10^ WM injury typically manifests as reduced WM volume and delayed myelination,^2^^,^^6^^,^^11^ though some studies describe increased WM volume in regions with reduced GM, reflecting maladaptive changes.^10^^,^^12^ Myelination impairments likely result from disrupted oligodendrocyte maturation driven by hypoxia and inflammation-induced microglial activation.^6^^,^^11^^,^^13^^,^^14^

Stem cell-based therapies can support brain development in the injured immature brain.^15^^,^^16^ Mesenchymal stem cells (MSCs) are particularly promising due to their neuroregenerative properties and favorable immune profile.^15–17^ In experimental models, MSCs act via paracrine mechanisms, releasing trophic and immunomodulatory factors that stimulate oligodendrocyte survival and maturation, reduce WM deficits, attenuate neuroinflammation, and improve functional outcomes.^15^^,^^18^ In experimental FGR models, MSCs have been shown to reduce neuroinflammation and oxidative stress, rescue neuronal survival, and improve functional outcomes.^19^^,^^20^ Despite growing clinical interest, stem cell therapies have not yet targeted newborns with FGR.^8^

Here, we evaluated intranasal delivery of human Wharton’s jelly-derived MSCs in the clinically relevant reduced uterine perfusion pressure (RUPP) rat model of chronic placental hypoperfusion-induced FGR. Building on our recent demonstration that this model recapitulates structural and functional brain deficits relevant to placental insufficiency-induced FGR infants^21^, we hypothesized that intranasal MSCs could ameliorate FGR-associated brain abnormalities. We assessed multiple postnatal treatment timepoints and assessed physiological, anatomical, and behavioral outcomes. This study is the first to investigate *intranasal* MSC therapy in FGR and evaluates its potential as a novel therapeutic strategy for targeting FGR-associated brain injury.

## Methods

### Ethical approval

This study was conducted according to Dutch and European regulations and approved by the local Animal Welfare Body (AWB, The Netherlands) under license AVD11500202010346 issued by the national competent authority (Centrale Commissie Dierproeven, CCD). All procedures were reported following the ARRIVE guidelines.

### RUPP model

Twenty-seven nulliparous and time-paired pregnant Sprague-Dawley rats (mean weight: 242 g; 11-12 weeks old) were obtained from Envigo (The Netherlands) and acclimated at the animal facility for 7-9 days (Utrecht University, The Netherlands). Chronic placental hypoperfusion was induced on embryonic day 14 (E14) using the RUPP model, resulting in placental insufficiency-induced growth restriction.^22^^,^^23^ On E14, dams were randomly assigned to sham (*n* = 10) or RUPP (*n* = 17), with baseline body weight balanced across groups.

Dams received carprofen (5 mg/kg, s.c.) at least 30 min before and again 24 h after surgery. Lidocaine (20 mg/kg, s.c.) was applied along the abdominal midline prior to incision. Surgery was performed on a heated surgical table under isoflurane anesthesia (induction 4%-5%, maintenance 2%-3%; ISOFLO®, Zoetis B.V., The Netherlands) in air/oxygen. Midline laparotomy exposed uterine horns, kept hydrated with warm saline. Fetal viability was noted before vessel manipulation. Uterine perfusion was reduced using silver foil clips (0.25 mm thickness, 99.95% purity; AG00-FL-000450, Goodfellow Cambridge Ltd., UK). One clip (1.5 cm × 1.5 mm × 0.2 mm [*L* × *W* × *⌀*]) was positioned around the lower abdominal aorta just above the iliac bifurcation, and 2 other clips (1.0 cm × 1.5 mm × 0.1 mm [*L* × *W* × *⌀*]) were placed on the right and left ovarian arteries to reduce compensatory perfusion. Sham-operated dams underwent identical surgery but with a single ovarian clip (1.0 cm × 1.5 mm × 0.1 mm [*L* × *W* × *⌀*]) in the peri-ovarian fat tissue without occluding vessels. After clip placement, uterine horns were returned to the abdominal cavity, and the abdominal wall was closed with separate absorbable sutures (#V451H, Johnson & Johnson, USA) for muscle and skin. Dams were housed individually after surgery and monitored twice daily to ensure normal recovery.

Pups were delivered at E21-23, and live births were documented. Day of birth was considered postnatal day 0 (P0). On P1, litters were culled to 7 pups, maintaining sex balance across treatment groups. RUPP pups were randomly allocated to (1) VEH, (2) P5 MSC, or (3) P10 MSC. To minimize litter effects, RUPP litters included different groups: histology experiment included pups from all experimental groups; behavioral experiments included VEH and P5-MSC pups only. Each pup was considered an independent experimental unit. Separate cohorts were performed for histology (surviving until P20) and behavioral testing (P60-61). Histological assessment at P20 was selected based on our previous characterization of the RUPP model showing robust structural brain alterations at this developmental stage.^21^ Histological and behavioral analyses were intentionally performed in separate cohorts to avoid potential confounding from repeated behavioral testing and training. Animal numbers and sex distribution are summarized in Table 1. Additional handling details are provided in Supplementary Materials and Methods and Table S1.

**Table 1.** Overview of animal numbers included in this study.

Experiment	Group	Litters	Males	Females	Total pups
**Histological analysis at P20**	sham	4	13	15	28
	VEH	9	10	9	19
	P5 MSC		11	10	21
	P10 MSC		10	10	20
**Behavior until P60-61**	sham	6	21	20	41
	VEH	8	13	13	26
	P5 MSC		12	15	27
**Total**	–	27	90	92	182

Reason for omission of any animal from a specific dataset is indicated in the corresponding figure legend, when applicable. Abbreviations: MSC, human mesenchymal stem cell-treated RUPP pups; P, postnatal day; RUPP, reduced uterine perfusion pressure; VEH, vehicle-treated RUPP pups.

### Intranasal MSC administration

MSCs were derived from Wharton’s jelly of the human umbilical cord, obtained from healthy donors after obtaining written informed consent.^24^ MSCs were provided by Chiesi Farmaceutici S.p.A, Italy. The detailed isolation procedure is patented under WO 2019/123407. Resulting MSCs fulfilled the International Society for Cell and Gene Therapy release criteria, with >90% of the cells expressing CD73, CD90, and CD105.^25^ Quality control testing confirmed the absence of bacterial, fungal, mycoplasma, and endotoxin contamination. MSC details and culture procedures are provided in the Supplementary Materials and Methods.

P5 and P10 were selected to represent earlier preterm and later term-equivalent postnatal intervention timepoints during ongoing brain development, reflecting relatively less and more mature stages of cortical and white matter maturation in the early postnatal brain^26–28^ These timepoints are clinically relevant given that placental insufficiency-induced FGR is frequently associated with preterm birth.^11^ RUPP pups received intranasal Dulbecco’s phosphate-buffered saline (DPBS) as vehicle (VEH; D8537, Merck KGaA) or MSCs at either P5 or P10 (dose: 50 × 10^6^ MSCs/kg of body weight; P5: 5 × 10^5^ MSCs per pup; P10: 9 × 10^5^ MSCs per pup). Half of the VEH pups received DPBS at P5, half at P10; and as no significant differences were detected between P5 and P10 VEH groups (Table S2), they were pooled and analyzed as a single RUPP-VEH group throughout the manuscript. Sham pups were untreated. 30 min prior to MSC or VEH administration, pups received nasal pretreatment with hyaluronidase (100 U in 12 µL; H4272, MilliporeSigma), delivered as 2 doses of 3 µL per nostril to enhance nasal mucosal permeability. After 30 min, MSCs or VEH were administered (P5: 2 doses of 3 µL per nostril [total 12 µL]; P10: 2 doses of 4.5 µL per nostril [total 18 µL]). Pups usually inhaled the drops spontaneously; if not, air was gently blown toward the face to elicit a startle reflex and promote uptake. Further details on immunohistochemistry, image acquisition and analysis, and behavioral assessments are provided in Supplementary Materials and Methods.

### Statistics

Sample sizes were calculated using G*Power v3.1^29^ based on prior RUPP effect sizes: 0.69 (90% power, one-tailed *α* = 0.05) for the immunohistochemistry experiment and 0.66 (90% power, one-tailed *α* = 0.05) for the behavioral outcome.

Data were analyzed using IBM SPSS Statistics v30.0.0.0 (IBM, USA). Outliers were identified by ROUT (*Q* = 1%) within each group (in GraphPad Prism v10.4.0, GraphPad Software, USA), and normality was assessed by Shapiro–Wilk test. A pre-defined sequential analytical approach was employed, with each step informed by the outcome of the previous one. One-tailed tests were applied based on our previous study showing RUPP-induced brain injury^21^ and the hypothesis that MSCs would ameliorate these deficits, producing unidirectional effects. First, model effect was evaluated by comparing sham and RUPP-VEH groups using a one-tailed unpaired *t*-test for parametric data with equal variances, Welch’s test for unequal variances, or Mann–Whitney *U* test for nonparametric data. If no significant model effect was observed, analysis was discontinued. If a significant model effect was identified, treatment effect was evaluated by comparing RUPP-VEH- and RUPP-MSC-treated pups (P5 or P10) using the same statistical criteria. Subsequently, when applicable, the two MSC treatments (P5 vs P10) were compared to observe whether timepoint influenced outcomes. Correlations were analyzed using Pearson’s correlation (parametric data) or Spearman’s rank correlation (nonparametric data). To assess whether pooling P5 and P10 vehicle-treated groups was justified, multivariate analyses of variance (MANOVA) were conducted for the primary outcomes (Table S2).

Data are depicted as boxplots (median [IQR], whiskers representing minimum and maximum values) for nonparametric data or as bar graphs (mean ± SEM) for parametric data, with all individual data points displayed. Statistical significance was set at *P *< .05.

## Results

### The RUPP procedure increases fetal resorption and reduces early postnatal body weight

**Figure 1.**
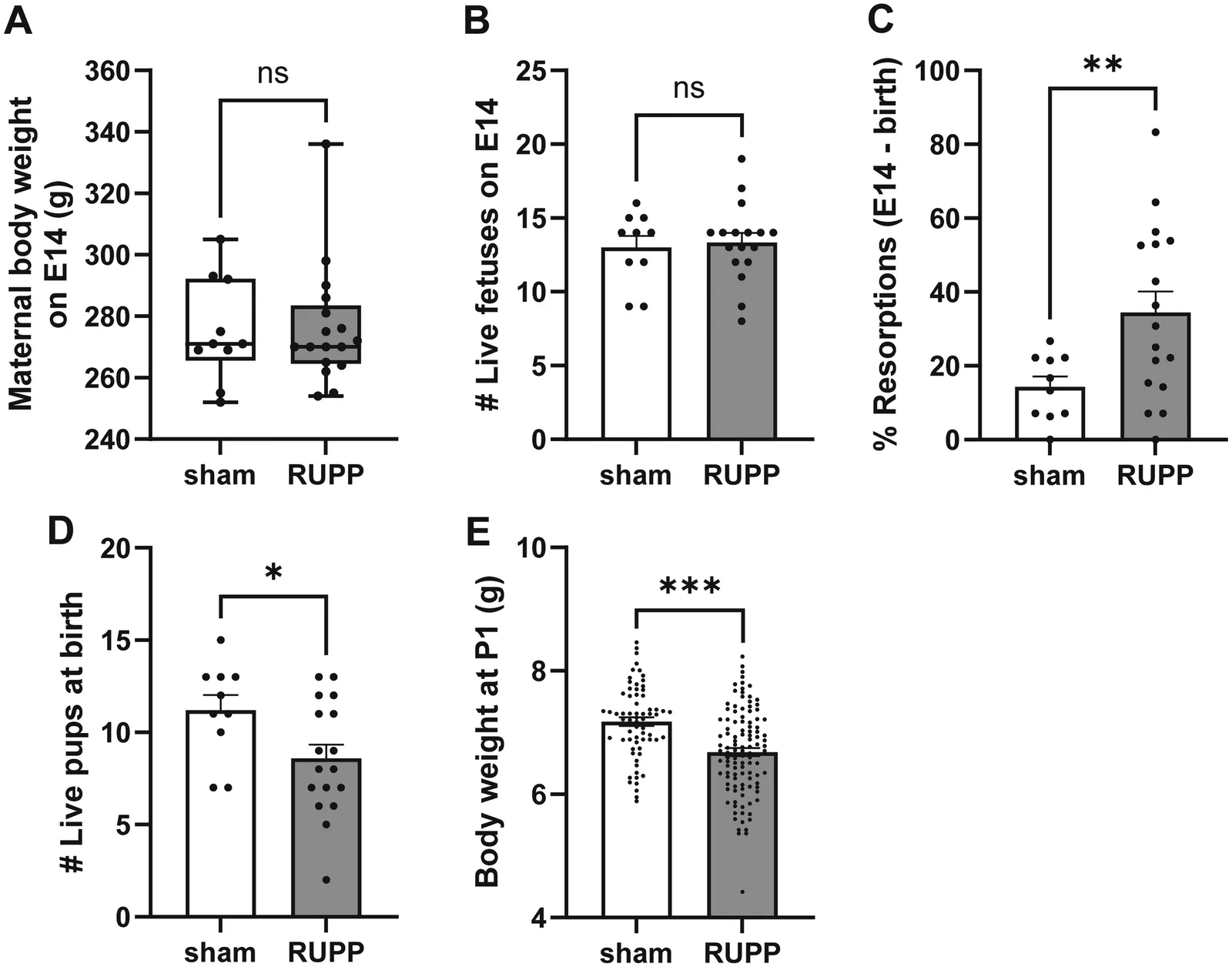
RUPP increases resorption rate, reduces live pups at birth, and decreases body weight at P1. Pregnant RUPP dams showed similar (A) maternal body weight and (B) number of live fetuses on E14, the day of RUPP procedure, compared to sham-operated dams. (C) RUPP increased fetal resorptions, (D) reduced live pups at birth, and (E) decreased body weight at P1. Comparisons were tested using a Mann–Whitney *U* test for nonparametric data (A) or an unpaired *t*-test for parametric data (B-E). Data are presented as median [IQR] (A) or mean ± SEM (B-E), as appropriate. *P < .05, **P < .01, ***P < .001. sham: *n* = 10; RUPP: *n* = 17. Sample sizes indicate the number of dams per group (A-D); panel E includes body weights of all included pups in the study (sham: *n* = 69; RUPP: *n* = 113). E, embryonic day; g, grams; ns, nonsignificant; P, postnatal day; RUPP, reduced uterine perfusion pressure.

Maternal body weight at E14 was similar between RUPP and sham dams (*P *= .490; Figure 1A), as was the number of live fetuses (*P *= .367; Figure 1B). The RUPP procedure increased fetal resorptions between E14-22 (*P *= .002, Figure 1C) with RUPP dams delivering fewer live pups (*P *= .017; Figure 1D). Pregnancy length did not differ between groups (sham: 22 [21.8-22.0] days, *n* = 10; RUPP: 22 [22-22] days, *n* = 17; *P *= .112). Fetal growth restriction was confirmed by a significant reduction in body weight at P1 in RUPP offspring compared to sham (*P *< .001; Figure 1E).

### Intranasal MSC treatment at P5 improves brain weight but not body weight in RUPP offspring

**Figure 2.**
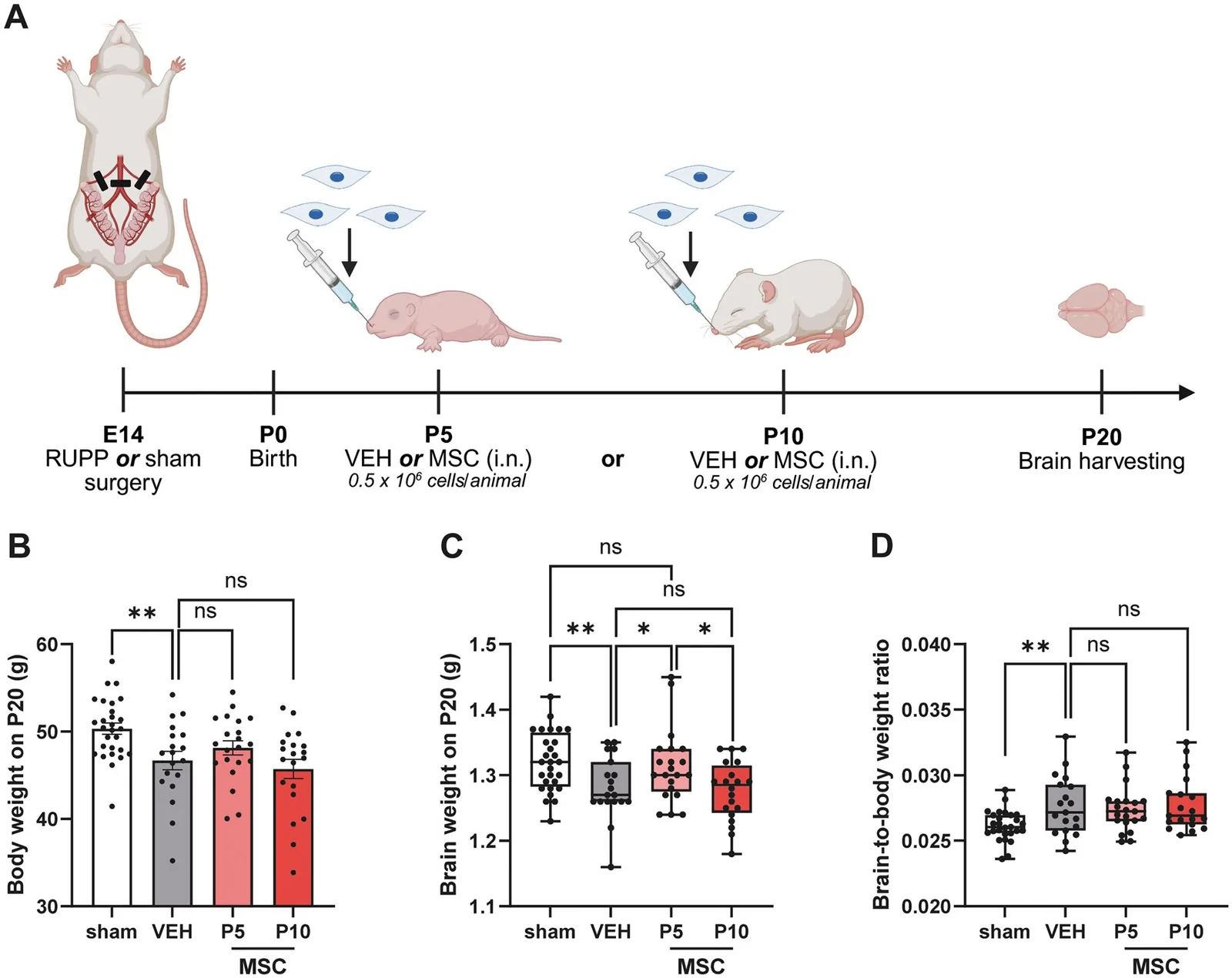
MSC treatment at P5 but not P10 improves brain weight in RUPP pups. (A) Timeline of the experimental design. Panel A was created using BioRender.com. (B) Body weight at P20 was reduced in RUPP-VEH pups compared to shams; MSCs at P5 or P10 did not improve body weight. (C) Brain weight was reduced in RUPP-VEH pups compared to sham pups and improved by P5 MSC treatment relative to RUPP-VEH but not P10; P5 vs P10 differed, with P5 treatment being more effective than P10. (D) Brain-to-body ratio was increased in RUPP-VEH pups compared to sham controls; MSCs had no effect. Comparisons were tested using an unpaired *t*-test for parametric data (B) or a Mann–Whitney *U* test for nonparametric data (C, D). Data are presented as mean ± SEM (B) or median [IQR] (C-D), as appropriate. *P < .05, **P < .01. sham: *n* = 28; VEH: *n* = 19; P5 MSC: *n* = 21; P10 MSC: *n* = 20. Panel D: two outliers (one sham, one MSC P10) were excluded from the analyses. Analyses including all observations did not alter statistical conclusions. Sample sizes indicate the number of pups per group. g, grams; MSC, human mesenchymal stem cell-treated RUPP pups; ns, nonsignificant; P, postnatal day; VEH, vehicle-treated RUPP pups.

RUPP pups were randomly assigned to receive MSCs or vehicle (VEH) at P5 or P10 (Figure 2A). Baseline body weights at P1 across all RUPP treatment groups were similar (Figure S1). Consistent with reduced P1 body weights, VEH-treated RUPP pups had lower body weight at P20 than sham controls (*P *= .002; Figure 2B). Intranasal MSC treatment at P5 or P10 did not significantly improve body weight (P5: *P *= .140; P10: *P *= .262; both vs VEH). In an additional cohort, body weight was monitored until P53, revealing no evidence of MSC-induced catch-up growth (Figure S2). RUPP-VEH pups had reduced brain weight compared to shams (*P *= .004; Figure 2C), which was attenuated by P5 MSC treatment (*P *= .048) but not by delayed P10 MSCs (*P *= .305), suggesting a potential beneficial effect specifically of early postnatal MSC therapy (*P *= .020; between P5 and P10). RUPP-VEH pups displayed an increased brain-to-body weight ratio compared to sham (*P *= .004; Figure 2D), indicative of brain-sparing, which was unaffected by MSCs (P5: *P *= .404; P10: *P *= .336; both vs VEH).

### Intranasal MSC treatment effectively attenuates increased cortical MBP immunoreactivity in RUPP rats

**Figure 3.**
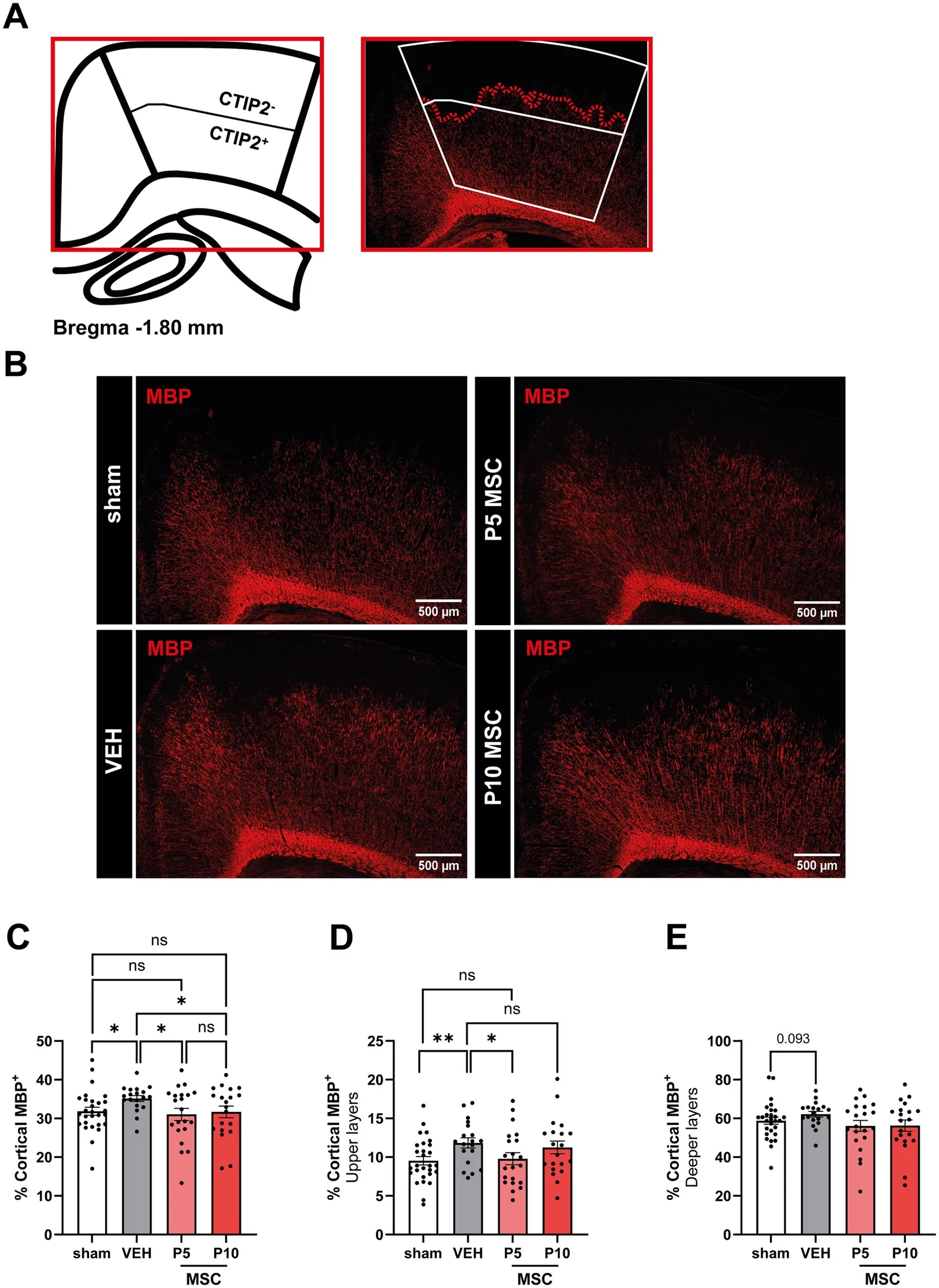
RUPP-induced aberrations in cortical myelination are attenuated by MSC treatment. (A) Schematic representation of image acquisition and cortical MBP^+^ analysis. (B) Representative MBP immunostainings images (2.5× magnification); contrast was enhanced for clarity in all images. (C) Cortical MBP^+^ area was increased in RUPP-VEH pups compared to sham pups and was reduced by MSC treatment at P5 or P10. (D) Increased MBP immunoreactivity was primarily observed in the upper cortical layers, with treatment effects observed only after MSC administration at P5 but not at P10. (E) No significant differences in myelination were observed in the deeper cortical layers between sham and RUPP-VEH pups. All comparisons were tested using an unpaired *t*-test (C-E). Data are presented as mean ± SEM. *P < .05, **P < .01. sham: *n* = 28; VEH: *n* = 19; P5 MSC: *n* = 21; P10 MSC: *n* = 20. Sample sizes indicate the number of pups per group. MSC, human mesenchymal stem cell-treated RUPP pups; MBP, myelin basic protein; ns, nonsignificant; P, postnatal day; VEH, vehicle-treated RUPP pups.

Cortical myelination, assessed by MBP immunoreactivity (Figure 3A and B), was increased in RUPP-VEH pups compared to shams (*P *= .012; Figure 3C), as observed in our previous study.^21^ Intranasal MSC treatments at P5 and P10 reduced MBP immunoreactivity compared to RUPP-VEH pups (P5: *P *= .012; P10: *P *= .023; both vs VEH; Figure 3C). Treatment timing did not affect efficacy (P5 vs P10: *P *= .388). The increase in MBP signal in RUPP pups was predominantly observed in the upper cortical layers (*P *= .005; VEH vs sham; Figure 3D), with no significant difference in the deeper layers (*P *= .093; VEH vs sham; Figure 3E). In the upper cortical layers, P5 MSC administration decreased MBP immunoreactivity compared to RUPP-VEH (*P *= .027; Figure 3D), while delayed P10 MSC treatment was ineffective (*P *= .296; P10 vs VEH; Figure 3D). Taken together, our findings in Figures 2 and 3 indicate that early P5 MSC treatment most effectively improved RUPP-induced brain abnormalities. Therefore, subsequent analyses focused on P5 MSC treatment only.

### Intranasal MSC treatment ameliorates loss of mature oligodendrocytes in the upper cortical layers after RUPP

**Figure 4.**
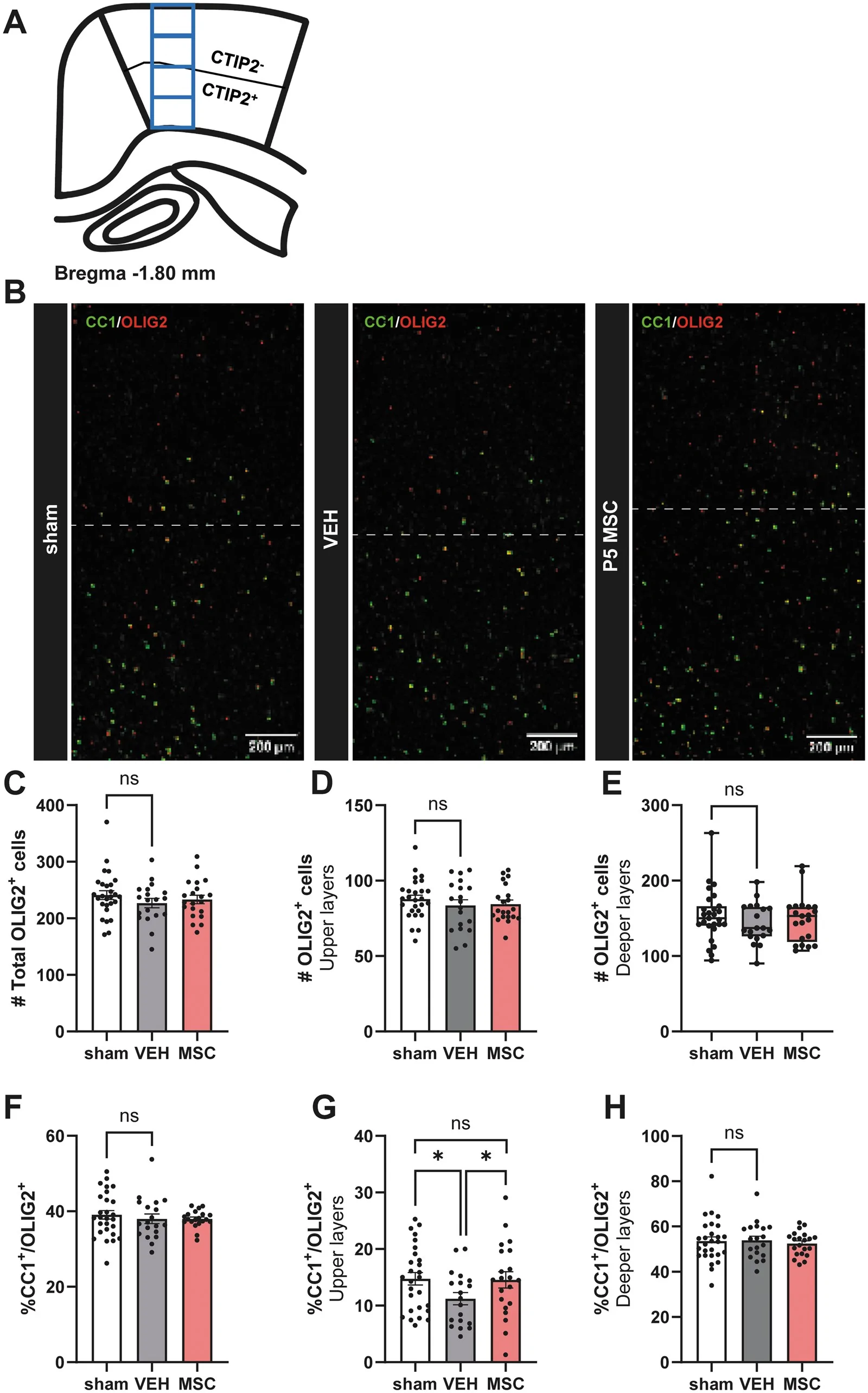
MSC treatment ameliorates loss of mature oligodendrocytes in the upper cortical layers after RUPP. (A) Schematic representation of image acquisition in the M2 cortex. (B) Representative stitched 10× images of CC1/OLIG2 co-labeling in the cortex (contrast adjusted individually per color channel for visualization). (C-E) Total OLIG2^+^ cell counts were unaffected by RUPP in total cortex (C), the upper (D) or the deeper cortical layers (E). (F) Ratio of CC1^+^/OLIG2^+^ cells were not affected in total cortex. (G) The upper cortical layers showed reduced CC1^+^/OLIG2^+^ ratio in RUPP-VEH pups, which was increased by MSC treatment relative to RUPP-VEH, reaching levels comparable to pups from sham-operated dams. (H) No differences in mature oligodendrocytes between groups were observed in the deeper cortical layers. Comparisons were tested using an unpaired *t*-test for parametric data (C-D, F-H) or a Mann–Whitney *U* test for nonparametric data (E). Data are presented as mean ± SEM (C-D, F-H) or median [IQR] (E), as appropriate. *P < .05. sham: *n* = 27; VEH: *n* = 19; MSC: *n* = 21; data from 1 sham animal (female) could not be assessed and therefore excluded from panels C-H. Panel F: one outlier (RUPP-MSC) was excluded from the analysis. Analysis including all observations did not alter statistical conclusions. Sample sizes indicate the number of pups per group. MSC treatment was intranasally administered at P5. CC1, adenomatous polyposis coli (APC) clone CC1; MSC, human mesenchymal stem cell-treated RUPP pups; ns, nonsignificant; OLIG2, oligodendrocyte transcription factor 2; P, postnatal day; VEH, vehicle-treated RUPP pups.

Total and mature oligodendrocytes in the cortex were assessed by OLIG2 immunostaining and CC1/OLIG2 co-labeling, respectively (Figure 4A and B). Total OLIG2^+^ cell numbers in the cortex, as well as separate counts in the upper and the deeper cortical layers, were unaffected by RUPP (VEH vs sham: total: *P *= .113; upper: *P *= .167; deeper: *P *= .160; Figure 4C-E). Overall, ∼40% of oligodendrocytes in the cortex were mature (CC1^+^/OLIG2^+^), with no difference between sham and RUPP pups (*P *= .227; Figure 4F). However, layer-specific analysis showed that RUPP reduced the percentage of CC1^+^/OLIG2^+^ cells in the upper cortical layers compared to sham (*P *= .017; Figure 4G), which was improved by MSC treatment (*P *= .038; MSC vs VEH). No significant differences in mature oligodendrocytes were observed in the deeper cortical layers between experimental groups (*P *= .454; VEH vs sham; Figure 4H).

### Intranasal MSC administration attenuates neuroinflammation in RUPP pups

**Figure 5.**
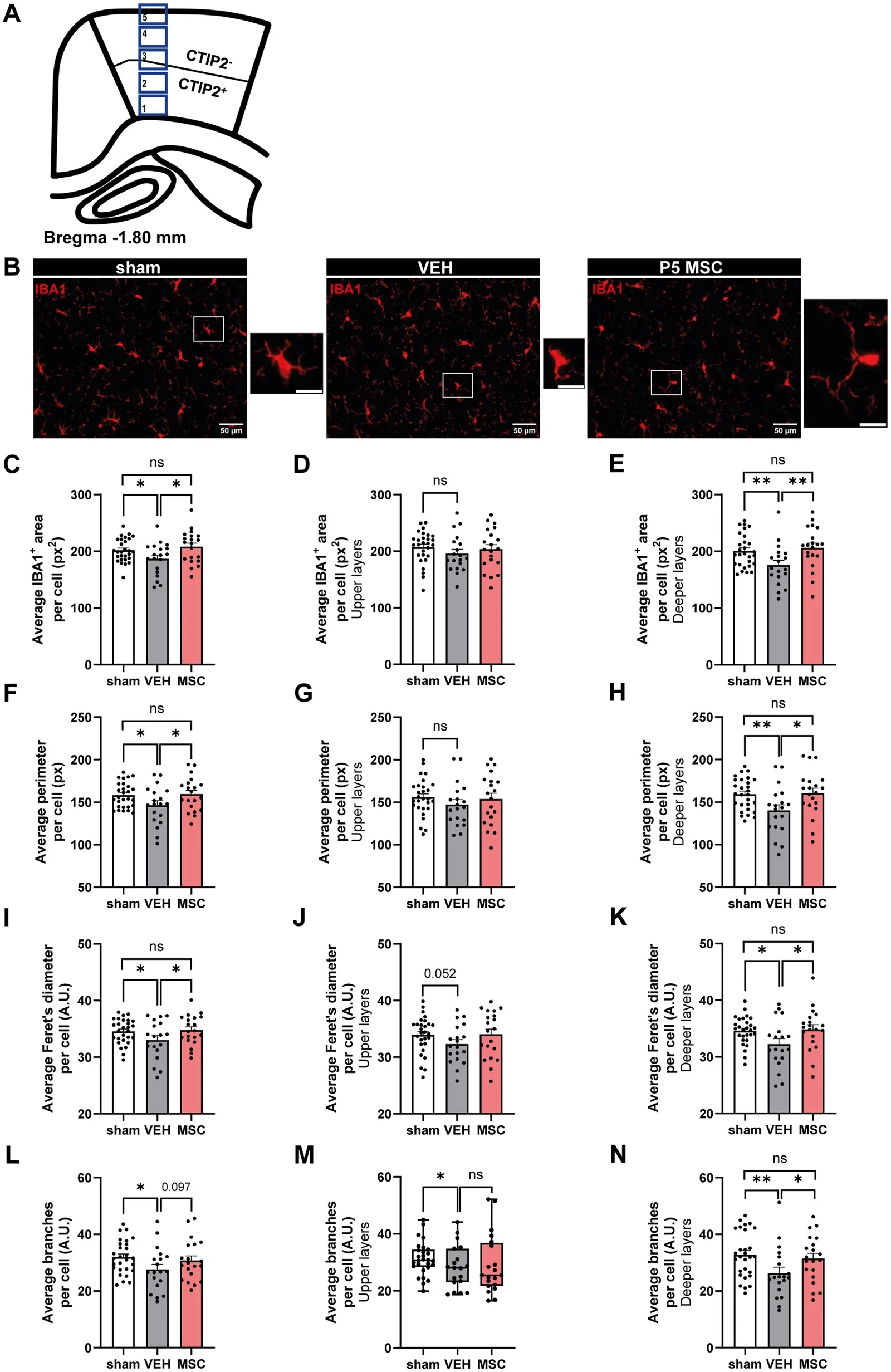
MSC treatment attenuates RUPP-induced changes in IBA1 morphology in the cortex. (A) Schematic representation of IBA1 image acquisition in the M2 cortex. Total cortical morphology: 20× images 1-4 averaged (image 5 occasionally contained areas without tissue). Layer-specific analyses: images 1-2 averaged for the deeper cortical layers, 4-5 for the upper cortical layers; image 3 (layer transition) excluded. (B) Representative 20× images of IBA1 immunostaining in the deeper cortical layers (scale insets: 15 µm); all images’ contrast enhanced for visualization. (C-E) Averaged IBA1^+^ area per cell was reduced in RUPP-VEH pups in total cortex (C), and the deeper cortical layers (E), indicating signs of microglial activation, and was ameliorated by MSC treatment; the upper cortical layers were unaffected (D). (F-H) Averaged perimeter per cell was reduced in RUPP-VEH pups in the total cortex (F) and the deeper cortical layers (H), and improved by MSCs; the upper cortical layers were unchanged (G). (I-K) Averaged Feret’s diameter per cell was decreased in RUPP-VEH pups in the total cortex (I) and the deeper cortical layers (K), with no changes in the upper cortical layers (J); MSC treatment improved levels to those observed in pups from sham-operated dams. (L-N) Average branches per cell were reduced in the RUPP-VEH pups in total cortex (L), the upper cortical layers (M), and the deeper cortical layers (N); MSC therapy significantly ameliorated branch number in the deeper cortical layers compared to RUPP-VEH. Comparisons were tested using an unpaired *t*-test for parametric data (C-L, N) or a Mann–Whitney *U* test for nonparametric data (M). Data are presented as mean ± SEM (C-L, N) or median [IQR] (M), as appropriate. *P < .05, **P < .01. sham: *n* = 28; VEH: *n* = 19; MSC: *n* = 20; 1 MSC animal (male) was excluded from the figure due to damaged cortex. Sample sizes indicate the number of pups per group. MSC treatment was intranasally administered at P5. A.U., arbitrary units; MSC, human mesenchymal stem cell-treated RUPP pups; IBA1, ionized calcium-binding adapter molecule 1; ns, nonsignificant; px, pixel; VEH, vehicle-treated RUPP pups.

Neuroinflammation in the cortex was assessed by IBA1 immunostaining (Figure 5A and B). RUPP did not alter IBA1^+^ cell numbers in the indicated total cortex area or when the upper/deeper cortical layers were separately analyzed (total: sham 118 [107-132] vs VEH 119 [97.3-130], *P *= .406; upper: sham 59.5 ± 2.23 vs VEH 59.4 ± 3.12, *P *= .482; deeper: sham 59.8 ± 1.52 vs VEH 61.5 ± 3.31, *P *= .305; sham: *n* = 28, VEH: *n* = 19). While IBA1^+^-cell numbers did not differ, RUPP-VEH pups showed signs of microglial activation, indicated by decreased IBA1^+^ area per cell (*P *= .029; VEH vs sham; Figure 5C), which was reversed by MSC administration (*P *= .014; VEH vs MSC). Layer-specific analysis showed no microglial activation in the upper cortical layers after RUPP (*P *= .113; Figure 5D), but a significant decrease in IBA1^+^ area per cell in the deeper cortical layers (*P *= .006; Figure 5E), rescued by MSC treatment (*P *= .007; VEH vs MSC).

Other morphological hallmarks, including total perimeter, Feret’s diameter (ie, distance between two parallel lines tangent to the cell body), and average number of branches of IBA1^+^ cells further supported signs of microglial activation in RUPP offspring^30–32^. Total IBA1^+^ perimeter, average Feret’s diameter, and average number of branches were reduced in the RUPP-VEH group in the total cortex compared to shams (*P *= .027; Figure 5F; *P *= .040; Figure 5I; *P *= .021; Figure 5L). MSC treatment improved the total perimeter (*P *= .032; Figure 5F) and average Feret’s diameter (*P *= .036; Figure 5I), while not reaching significance for the average number of branches (*P *= .097; Figure 5L). No significant activation of IBA1^+^ cells was observed based on morphological hallmarks in the upper cortical layers (*P *= .112; Figure 5G; *P *= .052; Figure 5J), except for the average of branches per cell, which was significantly reduced in the VEH group (*P *= .0497; Figure 5M). MSC administration did not improve the average number of branches per cell (*P *= .455). In the deeper cortical layers, RUPP significantly reduced the perimeter (*P *= .004; Figure 5H), Feret’s diameter (*P *= .028; Figure 5K), and number of branches (*P *= .007; Figure 5N) of IBA1^+^ cells compared to shams. MSCs significantly rescued the RUPP-induced changes in IBA1^+^ cell morphology in the deeper cortical layers (perimeter: *P *= .014; Figure 5H; Feret’s diameter: *P *= .034; Figure 5K; branches: *P *= .036; Figure 5N; all VEH vs MSC).

### Intranasal MSC treatment does not ameliorate neurobehavioral deficits in RUPP pups

**Figure 6.**
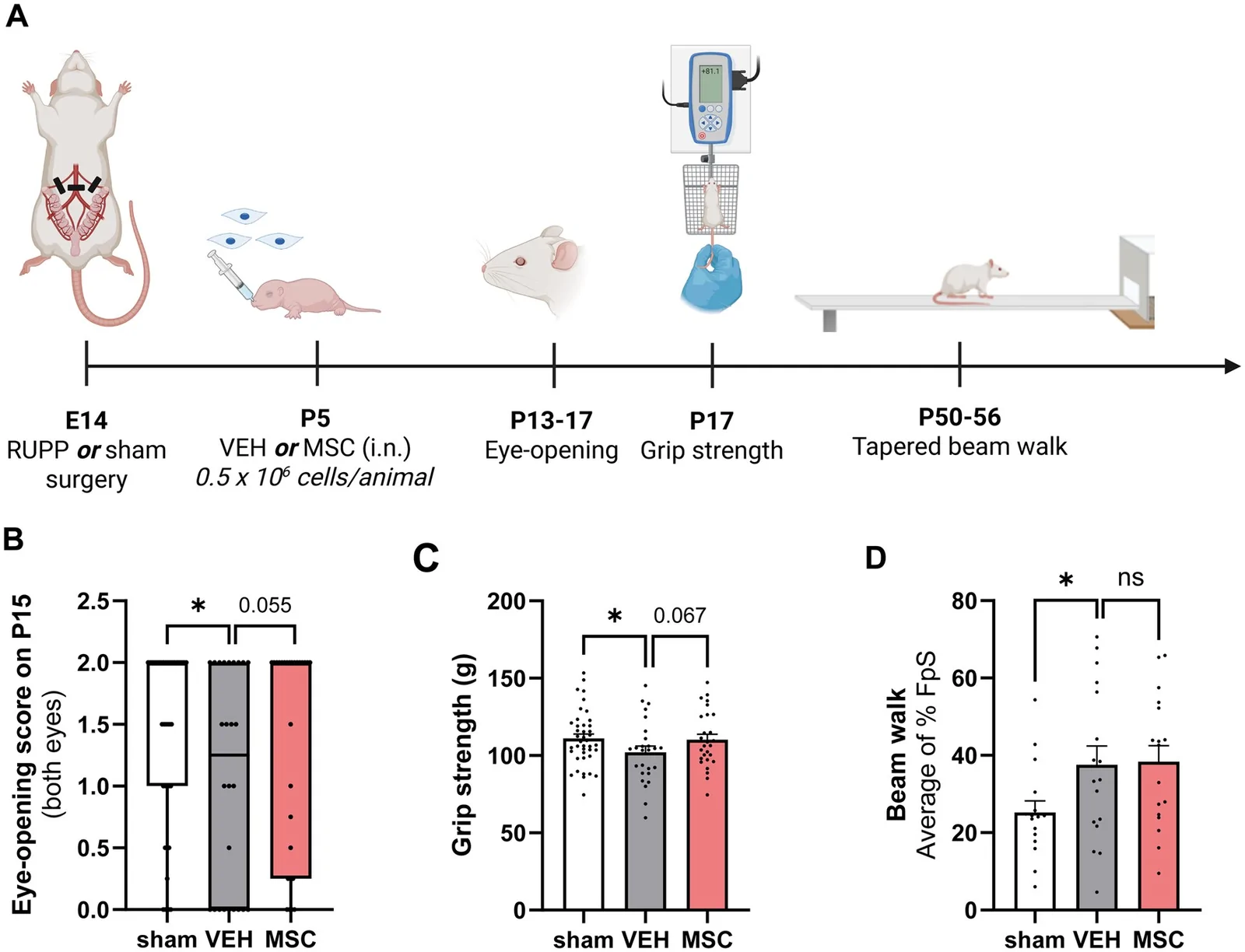
MSC treatment does not improve neurobehavioral outcome after RUPP. (A) Timeline of experiment and behavioral tests. Panel A was created using BioRender.com. (B) On the morning of P15, RUPP-VEH pups showed delayed eye-opening compared with sham pups; MSC treatment did not significantly improve the eye-opening score. (C) Grip strength was reduced in the RUPP-VEH group and was not significantly improved by MSC treatment. (D) In the tapered beam walk, RUPP-VEH animals showed increased average of foot-faults-per-step ratio; MSC treatment did not improve performance. Comparisons were tested using a Mann–Whitney *U* test for nonparametric data (B) or an unpaired *t*-test for parametric data (C, D). Data are presented as median [IQR] (B) or mean ± SEM (C, D), as appropriate. *P < .05. For panels B and C: sham: *n* = 41; VEH: *n* = 26; MSC: *n* = 27; for panel D: sham: *n* = 16; VEH: *n* = 17; MSC: *n* = 17. Due to time constraints on the number of animals that could be tested per day, 16-17 animals from each experimental group were tested in panel D. Sample sizes indicate the number of pups per group. E, embryonic day; FpS, foot faults per step; g, grams; MSC, mesenchymal stem cell-treated RUPP pups; i.n., intranasal administration; ns, nonsignificant; P, postnatal day; VEH, vehicle-treated RUPP pups.

Rats underwent three behavioral tasks during P13-P56 (Figure 6A). RUPP-VEH rats displayed delayed eye opening (*P *= .043; Figure 6B), reduced grip strength (*P *= .032; Figure 6C), and increased foot faults per step on the tapered beam walk (*P *= .020; Figure 6D), indicating impaired sensorimotor performance compared to shams. Intranasal MSC treatment at P5 did not significantly improve these outcomes, though some reached borderline significance (eye-opening: *P *= .055, Figure 6B; grip strength: *P *= .067, Figure 6C; beam walk: *P *= .453, Figure 6D; all VEH vs MSC). Grip strength correlated positively with body weight in shams (*P *= .001; Figure S3) but not in RUPP-VEH (*P *= .301) or RUPP-MSC (*P *= .352) pups, suggesting that reduced grip strength after RUPP is not attributable to lower body weight but rather reflects diminished muscular strength.

## Discussion

In this study, we showed that RUPP-induced prolonged uteroplacental hypoperfusion profoundly disrupted postnatal brain development, illustrated by reduced brain weight, altered cortical myelination, altered oligodendrocyte maturation, microglial activation, and functional impairments. Early postnatal (P5) intranasal MSC administration ameliorated several neuropathological alterations, despite no significant improvement in functional outcomes. This study highlights the translational relevance of the RUPP model for studying brain injury associated with chronic placental insufficiency-induced FGR and shows for the first time the potential therapeutic benefit of intranasally delivered MSCs to improve brain development after FGR.

Brain vulnerability associated with FGR overlaps with underlying hallmarks of other early-life brain injuries, including encephalopathy of prematurity, affecting, among other cell types, oligodendrocyte lineage cells and microglia. MSC therapy is of particular interest given its shown capacity to modulate neuroinflammation, restore oligodendrocyte maturation and myelination, and improve functional impairments across multiple neonatal brain injury models.^18^^,^^24^^,^^33–36^ Experimental FGR studies applying intravenous MSC delivery showed attenuation of cortical neuronal loss, reduced lumbar spinal cord hyperexcitability, and improvements in motor and social functional outcomes.^19^^,^^37^ The current study is the first to evaluate *intranasal* MSC therapy in the RUPP model of placental insufficiency-induced FGR.

In our study, intranasal MSC administration increased the reduced brain weight in RUPP offspring to levels comparable to pups from sham-operated dams, while body weight remained unaffected. Consequently, the brain-to-body weight ratio remained elevated, reflecting persistent brain-sparing, a compensatory response to chronic *in utero* hypoxia.

Additionally, RUPP offspring showed altered cortical myelination, which was attenuated by intranasal MSC administration at P5 or P10. In the upper cortical layers specifically, MSC treatment was effective only when administered at P5, suggesting that MSCs may be more capable of rescuing myelin formation during the onset phase of postnatal myelination.^26^^,^^38^ The observed elevated MBP levels despite fewer mature oligodendrocytes in the upper cortical layers suggest compensatory myelin overproduction, potentially resulting in poor axonal wrapping and disorganized myelin sheaths.^39–42^ Because MBP quantification alone does not assess in-depth myelin structural integrity, future electron microscopy studies should examine whether MSCs improve proper myelin sheath formation.

We hypothesize that maladaptive myelin production may be reinforced by neuroinflammation, as it has been shown that activated microglia promote dysfunctional oligodendrocyte maturation.^43^^,^^44^ MSC-induced attenuation of microglial activation may therefore underlie the observed histological improvements in our study. This aligns with findings from other FGR models in which intravenously delivered MSCs decreased the number of activated microglia^45^ and promoted anti-inflammatory phenotypes.^19^ MSCs are thought to exert their therapeutic effects by the release of trophic and immunomodulatory factors in the brain’s microenvironment.^33^^,^^46–48^ The Wharton’s jelly-derived MSCs used in this study secrete factors such as insulin-like growth factor 1 [IGF-1], glial cell-line derived neurotrophic factor [GDNF], brain-derived neurotrophic factor [BDNF], and basic fibroblast growth factor [bFGF].^24^ The MSC secretome, and these factors specifically, have been shown to reduce microglial activation and support oligodendrocyte progenitor survival, proliferation, differentiation, and myelin formation in experimental models of neonatal brain injury.^24^^,^^33^^,^^49^ Accordingly, we hypothesize that the MSCs’ paracrine mechanisms may underlie the beneficial effects observed in our study.

Despite anatomical and cellular improvements, behavioral assessments revealed no significant benefits of MSC treatment on functional outcome. Motor and neurodevelopmental tests were selected based on our earlier data showing these domains to be affected in RUPP offspring.^21^ The absence of functional improvements contrasts with other neonatal brain injury models, in which intranasal MSC therapy improved motor and cognitive outcomes.^33^^,^^36^^,^^50^ In experimental FGR studies, intravenously administered MSCs improved negative geotaxis, rotarod performance and produced modest, sex-specific improvements in social behavior.^19^^,^^37^ Lack of behavioral benefits in our study may reflect suboptimal MSC timing or dosing. Additionally, the tests used may not have been sensitive enough to detect subtle functional improvements, as suggested by borderline significant differences in grip strength and eye opening. Future studies should employ more sensitive, domain-specific tasks (eg, single pellet reaching test or ladder rung walking test) to better assess subtle functional impairments and further optimize MSC strategies to maximize therapeutic potential.

Variability in MSC efficacy across FGR models and underlying differences in functional outcomes is likely influenced by differences in developmental stage at the time of both insult and intervention. The timing of injury determines the affected developmental processes, while the timing of intervention influences the therapeutic window to effectively inhibit inflammation and promote repair. In FGR models induced at E17, intravenous MSC administration at P1-P4 (ie, ∼6-10 days post-induction) improved neuronal survival, lumbar spinal cord excitability, and functional performance.^19^^,^^37^ In the current study, intranasal MSC administration at P5 corresponds to ∼13 days post-start-of-insult, which may limit the therapeutic benefit compared with a reduced interval between insult and intervention. A shorter latency between onset of injury and MSC treatment may more effectively attenuate the evolving inflammatory cascade and preserve developmental trajectories during critical windows of cortical circuit assembly disrupted by the E14 insult, which are unlikely to be fully rescued at later stages.^51^ In addition, differences in the route of MSC administration may influence biodistribution and homing patterns, thereby shaping the mechanisms by which MSCs exert their therapeutic effects. MSC dose may also affect therapeutic efficacy. As the first study to employ intranasal MSCs in the RUPP model, we selected a dose within the effective range reported in other studies (0.5 × 10^6^ MSCs at P5-P9 in rodents).^33^^,^^52–54^ Although our selection represents the lowest effective dose in other models, a higher dose may be required in the RUPP model, particularly given the prolonged interval of 13 days between insult and start of treatment. In line, a previous study from our group in a preterm diffuse WM injury mouse model induced at P5 demonstrated that intranasal MSC treatment effectively treated diffuse WM injury when administered at 3 days post-insult, but not at 6-10 days,^33^ highlighting the importance of early postnatal intervention or a higher dose in neonatal brain injury models. In addition, given the transient survival of MSCs following administration,^50^ a single dose may not provide sustained therapeutic effects over the prolonged disease course in FGR. Repeated dosing during the early postnatal period may therefore represent a more effective strategy to enhance and prolong neuroprotective effects. Future optimization strategies, such as MSC preconditioning^55^ or combination therapies,^45^ may further enhance the efficacy of MSC treatment.

Although this study provides new insight into the therapeutic potential of MSCs for placental insufficiency-induced FGR, several limitations should be acknowledged. First, a single MSC dose was tested, limiting insight into optimal dosing. Although we and others have shown that intranasal MSC application predominantly targets the brain in other models of (neonatal) brain injury,^24^^,^^33^^,^^50^^,^^52^^,^^55–62^ MSC migration and biodistribution following intranasal administration have not yet been characterized in the RUPP model. Regional MSC distribution, persistence, and potential off-target accumulation remain, therefore, currently unknown. Future studies with quantitative tracking and region-specific analyses are needed to optimize MSC efficacy. Furthermore, intranasal administration was chosen to maximize brain targeting and minimize systemic effects but may limit potential therapeutic effects in peripheral organs affected by FGR. In addition, sham pups did not receive intranasal treatment. Although hyaluronidase is not expected to affect the observed outcomes, the absence of treatment should be considered when interpreting comparisons of RUPP groups with sham controls. Next, histological analyses were limited to P20 at one brain level and region. P20 was deliberately selected based on our previous characterization of the RUPP model demonstrating robust structural brain alterations at this developmental stage^21^ and because it coincides with rapid postnatal white matter maturation and myelin accumulation,^38^ providing a sensitive window to assess treatment effects on oligodendrocyte maturation and myelination. Nevertheless, assessment at a single histological timepoint does not establish whether the observed treatment effects persist into adulthood and may have missed effects at other developmental stages or in other brain regions. While both sexes were included, the study was not powered to detect sex-specific differences. A priori hypothesis-driven statistical analyses using sequential and one-tailed testing may increase the risk of type I error compared to more conservative approaches. In addition, litter could not be modeled as a random factor due to the limited number of litters, preventing estimation of between-litter variance.

This study also has several strengths. The RUPP model is clinically relevant, enhancing the translational relevance of our findings to FGR resulting from chronic uteroplacental hypoperfusion. Human MSCs were used, representing a therapeutic product suitable for future clinical use, thereby supporting clinical applicability. This is the first study to evaluate *intranasal* MSC delivery in FGR, highlighting a minimally invasive, clinically feasible route. Including both sexes and multiple litters improves generalizability and reduces potential bias, while comprehensive physiological, histological, and behavioral assessments provide an integrated assessment of cellular, anatomical, and functional outcomes.

## Conclusion

Postnatal intranasal MSC administration at P5 improves key neuropathological features of chronic placental insufficiency-induced FGR, including brain weight, oligodendrocyte maturation, myelination, and microglial activation. Functional recovery was not observed following MSC therapy, underscoring the importance of optimizing MSC timing, dosing, and delivery as well as the refinement of behavioral assessments. Future studies, including optimization strategies (eg, MSC preconditioning) or combination therapies, may enhance efficacy and further increase the clinical potential of MSC therapy. Overall, these findings provide a basis for further optimization of intranasal MSC therapy for placental insufficiency-induced FGR, with the ultimate aim of enhancing neurodevelopmental outcome in affected neonates.

## Supplementary Material

szag081_Supplementary_Data

## Data Availability

The data and materials supporting the findings of this study are available from the lead contact upon request. For additional information or requests for resources and reagents, please contact C.N. (c.nijboer@umcutrecht.nl).
