# Supplementary material for: Intranasal stem cell treatment ameliorates neuroinflammation and myelin aberrations in fetal growth-restricted rat pups
Source: Stem Cells Transl Med. 2026 Sep 12;15(9):szag081. doi: 10.1093/stcltm/szag081 (PMC13570797; doi:10.1093/stcltm/szag081)
Supplement: Supplementary file 1 [file szag081_supplementary_data.docx]

**SUPPLEMENTARY INFORMATION**

**Intranasal stem cell treatment ameliorates neuroinflammation and myelin aberrations in fetal growth-restricted rat pups**

**Running title:** Nasal MSCs improve neurological outcome after FGR

Judit Alhama-Riba^1^, Anastasija Aleksić^1^, Nicola Pelizzi^2^, Freek E. Hoebeek^1^, Caroline G.M. de Theije^1^, Cora H.A. Nijboer^1^

^1^Department for Developmental Origins of Disease (DDOD), Brain Center, University Medical Center Utrecht, Wilhelmina Children’s Hospital, Utrecht University, Utrecht, The Netherlands

^2^Global R&D, Chiesi Farmaceutici S.p.A., Parma, Italy

**Author contributions:**

**J.A.R:** conception and design, administrative support, collection and assembly of data, data analysis and interpretation, methodology, manuscript writing (original draft and review), final approval of manuscript

**A.A.:** collection and assembly of data, data analysis and interpretation, methodology, final approval of manuscript

**C.d.T.:** conception and design, administrative support, data interpretation, methodology, provision of study material, writing manuscript (review), final approval of manuscript

**N.P.**: provision of study material, final approval of manuscript

**F.H.:** conception and design, administrative support, data interpretation, provision of study material, final approval of manuscript

**C.N.:** conception and design, administrative support, financial support, data interpretation, methodology, provision of study material, writing manuscript (review), final approval of manuscript

**Corresponding author:** Cora H.A. Nijboer, PhD, Department for Developmental Origins of Disease, Wilhelmina Children’s Hospital, Lundlaan 6, 3584EA, Utrecht, The Netherlands. Telephone number: +31 88 755 4360; Email address: [c.nijboer@umcutrecht.nl](mailto:c.nijboer@umcutrecht.nl)

**Funding:** this work was founded by the European Union’s Horizon 2020 Research and Innovation program PREMSTEM under Grant Agreement No. 874721 (to CHAN).

**Table S1. Overview of primary and secondary antibodies used in the study.**

| **Antibody** | **Catalogue number** | **Manufacturer** | **Dilution** | **Target** |
| --- | --- | --- | --- | --- |
| **Primary antibodies** | | | | |
| Rat anti-CTIP2 | ab00616-7.4 | Absolute antibody, UK | 1:1000 | Deeper cortical layers V and VI |
| Rabbit anti-MBP | ab218011 | Abcam, UK | 1:2000 | Myelin |
| Rabbit anti-OLIG2 | AB9610 | Chemicon, MilliporeSigma, USA | 1:500 | Oligodendrocyte-lineage cells |
| Mouse anti-CC1 | OP80 | Calbiochem, MilliporeSigma, USA | 1:300 | Mature myelinating oligodendrocytes |
| Rabbit anti-IBA1 | 019-19741 | Fujifilm Wako Chemicals, USA | 1:2000 | Microglial cells |
| **Secondary antibodies** | | | | |
| Goat anti-rat – Alexa Fluor 488 | A11006 | Invitrogen, ThermoFisher Scientific, USA | 1:500 | CTIP2 |
| Goat anti-rabbit – Alexa Fluor 568 | ab175471 | Abcam, UK | 1:500 | MBP |
| Goat anti-rabbit – Alexa Fluor 594 | A11012 | Invitrogen, ThermoFisher Scientific, USA | 1:500 | OLIG2, IBA1 |
| Goat anti-mouse – Alexa Fluor 488 | A11001 | Invitrogen, ThermoFisher Scientific, USA | 1:500 | CC1 |

^a^ CC1: adenomatous polyposis coli (APC) clone CC1; CTIP2: COUP-TF interacting protein 2; IBA1: ionized calcium-binding adapter molecule 1; MBP: myelin basic protein; OLIG2: oligodendrocyte transcription factor 2.

**Table S2. Multivariate analyses assessing differences between P5 and P10 vehicle-treated pups to justify pooling across experimental datasets.**

| **Figure** | **Variables included** | **Pillai’s Trace** | **F (df1, df2)** | ***P*-value** |
| --- | --- | --- | --- | --- |
| 2 | Body weight on P20  Brain weight on P20  Brain-to-body weight ratio | 0.031 | 0.160 (3, 15) | 0.922 |
| 3 | % Cortical MBP  % Cortical MBP (upper layers)  % Cortical MBP (deeper layers) | 0.228 | 1.478 (3, 15) | 0.261 |

^a^ MBP: myelin basic protein; P: postnatal day.

**Figure S1. Baseline body weights at P1 were similar across all RUPP treatment groups.** RUPP-VEH pups had lower body weight compared to sham controls at P1, whereas no differences were observed between animals later divided among RUPP-VEH and RUPP-MSC groups. Data are presented as mean ± SEM. ****P*<0.001. sham: n=69; VEH: n=45; P5 MSC: n=48; P10 MSC: n=20. g: grams; MSC: human mesenchymal stem cell-treated RUPP pups; P: postnatal day; VEH: vehicle-treated RUPP pups.

**Alt text:** Bar graph showing baseline body weight at postnatal day 1 in sham and RUPP experimental groups.

**Figure S2. MSC treatment does not rescue the persistent body weight deficit observed in RUPP-VEH pups (related to Figure 2).** RUPP-VEH pups of both sexes showed persistently lower body weight with no evidence of catch-up growth, and MSC treatment did not normalize body weight. Data are presented as mean ± SEM on a log_2_ scale. **P*<0.05, ***P*<0.01, ****P*<0.001; $*P*<0.05, $$*P*<0.01, $$$*P*<0.001. Symbols indicate comparisons between sham and RUPP-VEH groups. *P*-values were derived from two-way repeated measures ANOVA followed by Šidák’s post-hoc test. sham: n=21M, n=20F; VEH: n=13M, n=13F; MSC: n=12M, n=15F. F: females; g: grams; MSC: human mesenchymal stem cell-treated RUPP pups; Log_2_: base-2 logarithmic scale; M: males; P: postnatal day; VEH: vehicle-treated RUPP pups.

**Alt text:** Line graphs showing body weight across postnatal development in male and female sham, vehicle-treated RUPP, and MSC-treated RUPP pups.

**Figure S3. Grip strength reduction in RUPP-VEH pups is unrelated to reduced body weight (related to Figure 6).** A) Sham pups showed a positive correlation between grip strength and body weight. B-C) RUPP-VEH and MSC groups displayed no correlation between grip strength and body weight, suggesting that the reduced grip strength in the VEH group is unrelated to their lower body weight and instead reflects diminished muscular strength. Descriptive statistics are presented as correlation coefficients, calculated using Spearman’s rank correlation (ρ) for non-parametric data, as appropriate (A, C) or Pearson’s correlation (r) for parametric data (B). sham: n=41; VEH: n=26; MSC: n=27. g: grams; MSC: human mesenchymal stem cell-treated RUPP pups; P: postnatal day; VEH: vehicle-treated RUPP pups.

**Alt text:** Scatter plots of grip strength and body weight measured on postnatal day 17 in sham, vehicle-treated RUPP, and MSC-treated RUPP groups.

**SUPPLEMENTARY MATERIALS AND METHODS**

**Animals**

Dams were group-housed (2-3 per cage) in conventional cages (Eurostandard Type IV S, 210 x 375 x 480 mm [H x W x D], Tecniplast, USA) on woodchip bedding, enriched with a wooden chew stick, a plastic shelter, and paper tissues. Environmental conditions were maintained at 20-24°C and 45-65% humidity, under a 12 h light/dark cycle (lights on 7 AM-7 PM), with unrestricted access to water and food (breeding chow: V1124-703, rat/mouse maintenance chow: V1535-000; Ssniff Spezialdiäten GmbH, Germany).

To standardize litter size, 8 RUPP pups were cross-fostered to litters born on the same day within the same experimental group (sham or RUPP). One RUPP litter delivered only 6 pups and was left unchanged, as there were insufficient pups available from the same experimental group for cross-fostering that day. Pups were identified via subcutaneous Indian ink paw markings (Royal Talens, The Netherlands). All pups survived from birth to the end of the study, except for 3 RUPP pups (3/116, 2.6% mortality) and 1 sham pup (1/70, 1.4% mortality), which were found dead between P3 and P5.

**MSC**

Chiesi UC-MSC (TAN 31813 SCB #7, Chiesi Farmaceutici S.p.A., Italy), derived from healthy donors, were used. Master cell banks were cryopreserved at passage 2 and working cell banks at passage 4. MSCs used in this study were obtained from working cell banks (passage 4), and no further passaging or subculturing was performed prior to experimentation. For each experiment, a new vial from the same working cell bank was thawed and used.

Cell identity and purity were confirmed by the provider using gene expression and surface marker profiling. Freshly isolated MSCs of the primary cell bank expressed APCDD1, FLVCR2, and PPARG, and did not express 3G5. Quantitative gene expression was assessed by real-time quantitative PCR (qRT-PCR) in two independent cell populations (biological replicates), using β-actin as a housekeeping gene and ΔCt analysis to determine expression of APCDD1, FLVCR2, and PPARG. The primary cell bank used for these analyses was established and characterized in March 2017. The cell population was negative for epithelial cell adhesion molecule (EpCAM) and endothelial cells (CD31). MSCs expressed the surface markers CD105 (endoglin), CD73 (ecto-5’-nucleotidase), and CD90 (Thy-1), and no telomerase activity was detected.

Cells met predefined acceptance criteria, including a yield of 20 × 10^6^ cells per vial, viability of ≥80%, and 80-90% confluency within 48 h of culture. In addition, cells displayed morphology consistent with stem cells, with the majority appearing small, spindle-shaped, and organized in defined colonies.

**MSC culture**

MSCs were thawed and resuspended in complete medium consisting of proprietary basal medium (07-0011, Lonza, USA) supplemented with 5% human platelet lysate (00920178, Lonza) and heparin (2000 U/L, H3149-10KU, MilliporeSigma, USA), filtered using a Quick Release Vacuum Filtration System (S2GPU05RE, Merck KGaA, Germany). Following resuspension, cells were centrifuged (500 × g, 10 min, room temperature), and the resulting pellet was resuspended in complete medium. Cells were counted (NucleoCounter NC-200, Chemometec, Denmark) and seeded in T75 culture flasks (1.125 × 10^6^ cells/flask; 430641, Corning, USA) containing complete medium. Cultures were maintained for 48 h in an incubator (37°C, 5% CO2, 95% humidity). After 48 h of culture, medium was removed from the flasks, and cells were washed twice with basal medium and subsequently trypsinized for 3-5 min using trypsin-EDTA 0.5% (3U/mL; 15400054, ThermoFisher Scientific, USA). Trypsin was neutralized with cold basal medium, and cell suspension was collected in tubes and centrifuged (300 × g, 10 min, 4°C). Supernatant was carefully aspirated, and cells were resuspended in cold basal medium. Cells from all flasks were pooled, filtered (40 μm; 352340, Corning), and counted. Thereafter the required amount of MSCs was centrifuged (300 × g, 10 min, 4°C), resuspended in cold DPBS (D8537, Merck KGaA) at the required concentration and stored on ice until in vivo administration (within 30 min).

**Immunohistochemistry**

At P20, pups were euthanized by an overdose of pentobarbital (250 mg/kg, intraperitoneally) and transcardially perfused with PBS followed by 4% paraformaldehyde (PFA) for fixation. After perfusion and decapitation, brains were harvested, the brainstem was cut as evenly as possible across all brains, and whole brains (i.e., cerebrum and cerebellum) were weighed on a high-precision scale (PCB 200-2, Kern & Sohn GmbH, Germany). Brains were then post-fixed for 24 h in 4% PFA at 4°C, and thereafter dehydrated in increasing ethanol steps (30%-100%), and paraffin-embedded. Coronal 8 µm sections were cut at the level referred to as bregma -1.80 for adult rat (Paxinos and Watson, 2006) on Superfrost microscope slides (631-0108, VWR, USA). After rehydration of sections in decreasing ethanol steps, sections were heated to 95°C in sodium citrate buffer (10 mM, pH 6.0; 3580.1, Roth, Germany) for 15 min for antigen retrieval (20 min for Oligodendrocyte transcription factor 2 (OLIG2) and adenomatous polyposis coli (APC) clone CC1 (CC1) staining). For ionized calcium-binding adapter molecule 1 (IBA1) staining sections were washed with PBS (1×; BP3994, Fisher Scientific, USA). For all other stainings, sections were washed with PBS/0.05% Tween 20 (P1379, MilliporeSigma). Blocking was performed by incubating sections with 2% bovine serum albumin (A3059, MilliporeSigma) in PBS (1×; BP3994, Fisher Scientific) for IBA1 staining, or with 5% normal goat serum (88R-NG001, Biosynth, Switzerland) for all other stainings. Following blocking, sections were incubated overnight with primary antibodies (Table S1). The next day, sections were washed in PBS and incubated for 1 h at room temperature with the appropriate secondary antibodies (Table S1). Cell nuclei were counterstained for 5 min with 4′,6-diamidino-2-phenylindole (DAPI, 1:5000; D9542, MilliporeSigma) and mounted in FluorSave reagent (345789, MilliporeSigma).

**Image acquisition and analysis**

Researchers were blinded to the experimental groups during image acquisition and subsequent analysis. All fluorescent images were acquired primarily from the motor cortex of the left hemisphere. This region was selected a priori because of the pronounced motor deficits observed in the RUPP model (Alhama-Riba et al., 2026). Images were captured using an Axio Observer Z1 microscope with Zen Blue software and an AxioCam MRm camera (Carl Zeiss, Germany). All MBP, OLIG2 (co-labeled with CC1), and IBA1 stainings were co-labeled with CTIP2 to delineate the upper and the deeper cortical layers.

For myelin basic protein (MBP) analyses, images were taken at 2.5× magnification from a standardized cortical field of view that encompassed the secondary motor (M2), primary motor (M1), and the hindlimb region of the primary somatosensory (S1HL) cortices. MBP analyses were processed semi-automatically using a custom macro in Fiji (ImageJ) software v1.53q (Schindelin et al., 2012). For each image, the entire cortical area in the field of view and COUP-TF interacting protein 2 (CTIP2)-positive layers were manually outlined using the polygonal selection tool. From these outlines, the total cortical area, CTIP2^+^ area, and CTIP2^-^ area (calculated as total cortex minus CTIP2^+^) were obtained (see Figure 2E). Next, a manual threshold was applied to the MBP^+^ signal. Because the cortical and CTIP2^+^ regions had already been delineated, the percentage cortical area covered by MBP^+^ signal could be quantified both across the entire cortex in the field of view and within the upper (CTIP2^-^) and the deeper (CTIP2^+^) cortical layers. For MBP analyses, all outlining and thresholding settings were kept consistent across all images.

For cell counting, overlapping images of the M2 cortex were acquired at 10 × magnification and stitched in Fiji to quantify OLIG2^+^ cells, the proportion of OLIG2^+^ cells that co-express CC1 (CC1^+^/OLIG2^+^; see Figure 3E), and IBA1^+^ cells. Cells were counted using the Cell Counter plugin in Fiji. The total cortical thickness and area were first measured using the straight line and polygonal selection tools, respectively. For each cell, its Y-coordinate along the cortical thickness was compared to the CTIP2^+^ layer boundaries: cells located within the CTIP2^+^ layer were assigned to the deeper cortical layers, and cells outside this layer to the upper cortical layers. Based on these assignments, OLIG2^+^, CC1^+^/OLIG2^+^, and IBA1^+^ cells were quantified separately in CTIP2^+^- and CTIP2^-^- layers, and total counts were determined. IBA1^+^ cells were expressed relative to the analyzed area. DAPI counterstaining was used for cell inclusion criteria.

For microglia morphology analyses, 5 non-overlapping images at 20× magnification were acquired per animal in the motor cortex starting from the upper edge of the corpus callosum going outward (see Figure 5A). For the total cortical analyses, images 1-4 were averaged, as the most superficial picture (image 5) occasionally contained areas without tissue. For layer-specific analyses, images 1 and 2 were averaged to represent the deeper cortical layers, while images 4 and 5 were averaged to represent the upper cortical layers. Image 3 was located at the transition between the upper and the deeper layers, and was therefore excluded from layer-specific analyses. Microglial morphology was assessed using the particle analysis function in Fiji, following previously described methods (van Tilborg et al., 2018b; Zanier et al., 2015). Briefly, images were pre-processed with a custom macro to enhance contrast and intensity. IBA1^+^ signal was manually thresholded, and microglia were automatically selected using the ‘Analyze particles’ function with a surface range of 800-12,000 pixels. Cells were manually checked for the presence of DAPI^+^ nuclei and excluded if no nucleus was detected; any IBA1^+^/DAPI^+^ cells missed by the ‘Analyze particles’ function were manually added. Images were then converted to binary format and skeletonized. For each cell, IBA1^+^ area, perimeter, Feret’s diameter diameter, and number of branches were measured, and these values were averaged across all images per animal to assess microglial activation status.

**Behavioral assessments**

Researchers were blinded to the experimental groups during behavioral testing and scoring. Rats were habituated to the experimental room prior to behavioral testing to reduce stress and minimize environmental novelty effects. All behavioral tasks were conducted during the light phase and under standard light conditions. To minimize potential influences of circadian rhythm, each task was performed at a consistent time of day across all testing days. The selected behavioral paradigms focused on evaluating motor function.

For neonatal behavioral assessments (eye-opening and grip strength), all littermates were tested consecutively to avoid prolonged disturbance of the litter, with the testing order randomized within each litter. The daily order in which litters were evaluated was also randomized to prevent order effects across testing days. For the tapered beam walk, the testing sequence was randomized and counterbalanced across experimental groups and sexes, with males and females tested in alternating order. To prevent order effects across days, cages were tested in blocks (groups of cages tested consecutively), and the order of these blocks was rotated on consecutive testing days. The beam was cleaned with 5% ethanol in water between animals.

Rats were weaned between P26 and P29. Males and females were separately housed with same-sex littermates in groups of 3-4 animals. On three occasions, when a same-sex littermate was not available, an animal was housed with 2-3 non-littermates of the same sex.

**Eye-opening**

Eye-opening represents a key developmental milestone associated with visual cortex maturation and is considered indicative of early neurodevelopmental trajectories (Nguyen et al., 2017). A modified version of the procedure described by Nguyen and colleagues (Nguyen et al., 2017) was employed. Briefly, between P13 and P17, each pup’s eyes were examined twice daily at fixed time points (10:00 AM and 3:00 PM). Each eye was scored using the following scale: 0=closed eyelid, 0.5=partially open, and 1=fully open. The total eye-open score corresponded to the sum of both eyes, and results show the score measured on the morning of P15. A delay in eye-opening was interpreted as a potential indicator of impaired neurodevelopment.

**Grip strength**

Grip strength is a commonly employed measure of muscle function, motor coordination, and strength (Munier et al., 2022). At P17, each pup was weighed and subjected to a minimum of five consecutive trials. During each trial, once the pup grasped the bar handle, its tail was gently pulled, and the maximal peak force per trial was measured using a grip strength meter (BIP-GS3, Bioseb, France). Results show the average maximal peak force across the first three trials, which best represents each pup’s maximal grip strength. Reduced grip strength is indicative of neuromuscular impairment.

**Tapered beam walk**

The tapered beam walk test was used to evaluate bilateral motor coordination and balance in adult rats, using a modified protocol based on Brandt and colleagues (Brandt et al., 2024). P50-56 rats were trained and tested on an elevated tapered beam measuring 175 cm in length, 2 cm in thickness, and with the width tapering from 6 cm at the start to 1.5 cm at the end. A dark goal box containing bedding, a plastic shelter, and paper tissues was positioned at the narrow end of the beam. To remove olfactory cues, the tapered beam was cleaned with a 5% ethanol solution in demineralized water between animals.

The 4-day protocol consisted of 2 consecutive days of training followed by 2 consecutive days of testing. On the first training day and both testing days, a safety ledge protruding for 2 cm on either side of the beam was attached beneath it. The ledge was removed on the second training day to encourage unassisted beam traversal. Each training day began with a 2 min habituation in the dark box, followed by 5 consecutive runs per animal starting from the wide end of the beam. After each run, rats remained in the dark box for 1 min. By the end of the second training day, all animals were able to traverse the beam without pausing.

On each testing day, each rat performed 4 runs, with the last 3 runs recorded on video and used for analysis, resulting in a total of six testing runs per animal. Analyses focused on the central 120 cm of the beam, excluding the first 30 cm and the final 25 cm. Videos were manually scored to quantify foot slips.

Results are reported as the average of the foot-faults-per-step ratio across all paws and testing runs. For each testing run, the number of foot faults per paw was scored (half-slips = 0.5 and full-slips = 1) and divided by the number of steps taken by that paw. Values from all four paws were then summed, and the mean across the six testing runs per animal was calculated to yield the final foot-faults-per-step ratio. A higher ratio indicates impaired motor coordination and balance.
